# An evaluation of the effects of exogenous ethephon, an ethylene releasing compound, on photosynthesis of mustard (*Brassica juncea*) cultivars that differ in photosynthetic capacity

**DOI:** 10.1186/1471-2229-4-21

**Published:** 2004-12-30

**Authors:** NA Khan

**Affiliations:** 1 Department of Botany, Aligarh Muslim University, Aligarh 202 002, India

## Abstract

**Background:**

The stimulatory effect of CO_2 _on ethylene evolution in plants is known, but the extent to which ethylene controls photosynthesis is not clear. Studies on the effects of ethylene on CO_2 _metabolism have shown conflicting results. Increase or inhibition of photosynthesis by ethylene has been reported. To understand the physiological processes responsible for ethylene-mediated changes in photosynthesis, stomatal and mesophyll effects on photosynthesis and ethylene biosynthesis in response to ethephon treatment in mustard (*Brassica juncea*) cultivars differing in photosynthetic capacity were studied.

**Results:**

The effects of ethephon on photosynthetic rate (P_N_), stomatal conductance (g_S_), carbonic anhydrase (CA) activity, 1-aminocyclopropane carboxylic acid synthase (ACS) activity and ethylene evolution were similar in both the cultivars. Increasing ethephon concentration up to 1.5 mM increased P_N_, g_S _and CA maximally, whereas 3.0 mM ethephon proved inhibitory. ACS activity and ethylene evolution increased with increasing concentrations of ethephon. The corresponding changes in g_s _and CA activity suggest that the changes in photosynthesis in response to ethephon were triggered by altered stomatal and mesophyll processes. Stomatal conductance changed in parallel with changes in mesophyll photosynthetic properties. In both the cultivars ACS activity and ethylene increased up to 3.0 mM ethephon, but 1.5 mM ethephon caused maximum effects on photosynthetic parameters.

**Conclusion:**

These results suggest that ethephon affects foliar gas exchange responses. The changes in photosynthesis in response to ethephon were due to stomatal and mesophyll effects. The changes in g_S _were a response maintaining stable intercellular CO_2 _concentration (C_i_) under the given treatment in both the cultivars. Also, the high photosynthetic capacity cultivar, Varuna responded less to ethephon than the low photosynthetic capacity cultivar, RH30. The photosynthetic capacity of RH30 increased with the increase in ethylene evolution due to 1.5 mM ethephon application.

## Background

Photosynthesis is controlled by several intrinsic and extrinsic factors. Of these, plant hormones have received considerable attention in the past in photosynthetic responses of plants. Ethylene is a phytohormone that influences every aspect of plant growth and development [1]. It is synthesized by the activity of 1-aminocyclopropane carboxylic acid synthase (ACS). The response of plants to ethylene depends on the sensitivity of plants to the gas. Conflicting results on the effects of ethylene-releasing compounds on net photosynthetic rate (P_N_) have been reported. It has been shown to increase P_N _[2-7] or decrease it [8,9], but no definite reason has been assigned for this. It has been shown that the increase in P_N _with ethylene-releasing compounds was due to the increase in chlorophyll per unit leaf area [10] or by greater light interception [11]. In my earlier report it has been shown that alteration in photosynthesis was due to the changes in ACS activity [12]. The goal of this work was to compare stomatal and mesophyll effects on P_N _in response to ethephon treatment. For that, P_N_, stomatal conductance (g_S_) and carbonic anhydrase (CA) activity were recorded. To find a possible relationship of ethylene-mediated changes in foliar gas exchange parameters, activity of ACS and ethylene evolution were also determined. The work was carried out in two cultivars of mustard previously shown to have different photosynthetic capacity [12].

## Results

**Figure 1:**
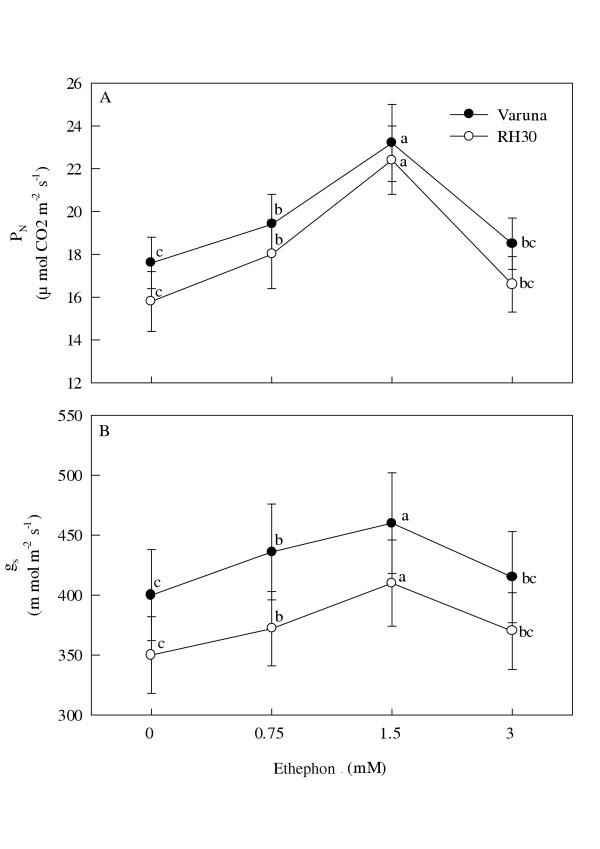
**Effects of ethephon on photosynthesis and stomatal conductance in mustard **Effect of different concentrations of ethephon (2-chloroethyl phosphonic acid) applied at 30 d after sowing on net photosynthetic rate (P_N_) (A) and stomatal conductance (g_S_) (B) in high photosynthetic capacity cultivar Varuna and low photosynthetic capacity cultivar RH30 of mustard (*Brassica juncea*) at 15 d after the treatment. Each data point represent treatment mean ± SE. Values at each data point within the cultivar sharing the same letter are not significantly different at P < 0.05.

**Figure 2:**
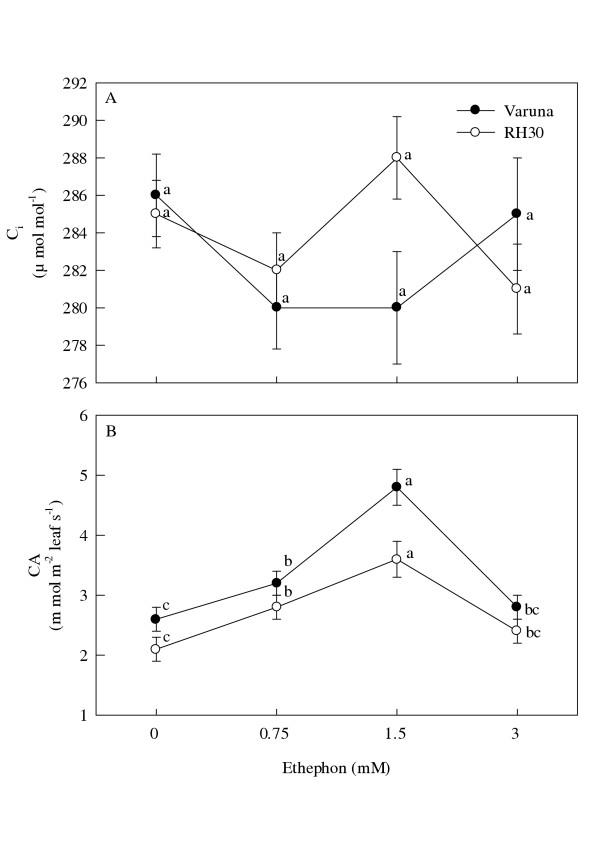
**Effects of ethephon on intercellular CO_2 _concentration and carbonic anhydrase activity in mustard **Effect of different concentrations of ethephon (2-chloroethyl phosphonic acid) applied at 30 d after sowing on intercellular CO_2 _concentration (C_i_) (A) and carbonic anhydrase (CA) activity (B) in high photosynthetic capacity cultivar Varuna and low photosynthetic capacity cultivar RH30 of mustard (*Brassica juncea*) at 15 d after the treatment. Each data point represent treatment mean ± SE. Values at each data point within the cultivar sharing the same letter are not significantly different at P < 0.05.

The effects of ethephon on P_N_, g_S _and CA were found significant in both the cultivars (Figures 1, 2). Ethephon at 1.5 mM increased the characteristics maximally, increasing P_N _by 31.8 and 41.8%, g_S _by 15.0 and 17.1% and CA by 84.6 and 71.4% in Varuna and RH30, respectively. Higher concentration of ethephon (3.0 mM) decreased the characteristics in both the cultivars. The ratio of intercellular to ambient CO_2 _concentration (C_i_/C_a_) was constant.

Ethephon application significantly affected ACS activity and ethylene evolution, and were greatest with 3.0 mM ethephon (Table 1). Low photosynthetic capacity cultivar, RH30 was more responsive to ethephon than the high photosynthetic capacity cultivar, Varuna. Application of 1.5 mM ethephon increased ethylene by 52.6% in Varuna and 75.0% in RH30. Increase in ethylene with 1.5 mM ethephon was associated with the increase in P_N_, g_S _and CA. Ethylene evolution with 3.0 mM ethephon proved inhibitory for photosynthetic parameters.

## Discussion

Maximum rates of photosynthesis were found with 1.5 mM ethephon. The increase in P_N _due to ethephon has been reported [4-6]. Increased g_s _and CA values in both the cultivars showed stomatal and mesophyll effects on photosynthesis. Mesophyll effects are characterized as a product of CO_2 _binding capacity and the electron transport capacity. The carboxylation capacity determines the mesophyll effects [13,14]. Increase in CA activity at the site of CO_2 _fixation exhibited the enhanced carboxylation reaction [15-17]. The changes in stomatal conductance due to ethephon were to maintain stable intercellular CO_2 _concentration (C_i_) under the given treatment. Thus, stomatal and mesophyll processes contributed to the increase in P_N _in response to ethephon. The ethephon-induced effects on photosynthetic parameters were mediated by ethylene evolved due to ethephon treatment. Taylor and Gunderson [18] showed a relationship between ethylene-enhanced g_S _and ethylene-enhanced P_N_. Higher concentration of ethephon (3.0 mM) decreased the P_N _and g_S_. Such condition of inhibition of P_N _by ethylene-releasing compound has been observed by Kays and Pallas [8] and Rajala and Peltonen-Sainio [9]. In all these studies ethylene has been attributed to the changes in P_N _due to its effect on g_S_. Mattoo and White [19] reported that ethylene affected CO_2 _assimilation and the plant responded depending on the tissue concentration. On the similar lines, Dhawan *et al*. [20], Kao and Yang [21] and Grodzinski *et al*. [22] reasoned that decrease in CO_2 _regulated P_N _and was related to ethylene evolution. In the present study, low photosynthetic cultivar, RH30 responded more to ethephon than the high photosynthetic cultivar, Varuna. In control plants, lesser ethylene evolution in RH30 than Varuna was responsible for lesser P_N_. As the ethylene evolution increased with ethephon application, the capacity of RH30 for P_N _also increased resulting in higher per cent increase in P_N _than the Varuna. An increase of 75% ethylene in RH30 due to 1.5 mM ethephon increased P_N _by 41.8%, whereas 52.6% increase in ethylene in Varuna due to the same treatment increased P_N _by 31.8%. Earlier strong positive correlation between ACS activity and P_N _has been shown [12].

It therefore, appears possible that the threshold value for ethylene with 1.5 mM ethephon was comparable to that which elicits the ethylene-mediated hormonal responses, which differ with the cultivars inherent capacity of physiological processes. It is that there is some requirement of ethylene for optimum response. Low and high concentration represent the two ends of an optimum curve, promoting at low concentration and inhibiting at high.

## Conclusions

This study shows that ethephon affects P_N _in both high and low photosynthetic capacity cultivars, Varuna and RH30. In both the cultivars, changes in P_N _were due to stomatal and mesophyll effects. Ethephon-induced P_N _was attributed to ethylene evolution. The high photosynthetic capacity cultivar, Varuna responded less to ethephon than the low photosynthetic capacity cultivar, RH30. The low P_N _of RH30 was due to low level of ethylene. The low photosynthetic capacity of RH30 could be enhanced to give higher P_N _through increase in ethylene evolution. However, for both the cultivars there is a range of physiologically active concentration of ethylene beyond which it exerts inhibitory effects.

## Methods

Two cultivars of mustard (*Brassica juncea *L. Czern & Coss.), namely Varuna (high photosynthetic capacity) and RH30 (low photosynthetic capacity) were grown from seeds in 10 m^2 ^field plots in complete randomized design with five replications. At seedling establishment a plant population of 12 plants m^-2 ^was maintained and recommended plant cultivation procedures were adopted. A uniform recommended soil application of 18 g N, 3 g P and 3 g K m^-2 ^was given at the time of sowing so as the nutrients were non-limiting.

At 30 d after sowing, 0, 0.75, 1.5 and 3.0 mM ethephon (2-chloroethyl phosphonic acid) was sprayed with a hand sprayer. Ethephon is a direct ethylene source when applied to plants and elicits response identical to those induced by ethylene gas [23,24]. Since ethephon on hydrolysis releases ethylene and phosphorus, therefore equivalent amount of phosphorus present in 3.0 mM ethephon was given to all treatments including control to nullify the effects of phosphorus.

At 45 d after sowing (15 d after ethephon treatment) P_N_, g_S_, C_i_, CA activity, ACS activity and ethylene evolution were determined.

### Measurement of photosynthetic parameters

P_N_, g_S _and C_i _were measured using infrared gas analyzer (LiCOR 6200, Lincoln, NE) on fully expanded upper most leaves at saturating light intensity on four plants from each replicate. The atmospheric conditions during the experiment between 1100–1200 h were: photosynthetic active radiation about 1050 μmol m^-2 ^s^-1^, relative humidity 64% and temperature 23°C, atmospheric CO_2 _concentration 360 μmol mol^-1^.

### Measurement of carbonic anhydrase activity

The leaves used for photosynthesis measurement were selected for CA activity determination. CA was measured by the method of Dwivedi and Randhava [25]. Leaves were cut into small pieces in 10 mL of 0.2 M cystein at 4°C. The solution adhering to the leaf surface was removed and immediately transferred to a tube having 4 mL phosphate buffer (pH 6.8). A 4 mL of 0.2 M sodium bicarbonate in 0.002 M sodium hydroxide and 0.2 mL of 0.002% bromothymol blue was added to the tube. The tubes were kept at 4°C for 20 min after shaking. Liberated CO_2 _during the catalytic action of enzyme on sodium bicarbonate was estimated by titrating the reaction mixture against 0.05 N hydrochloric acid.

### Measurement of ACS activity and ethylene evolution

Activity of ACS was measured adopting the methods of Avni *et al*. [26] and Woeste *et al*. [27]. Leaf tissue was grind in 100 mM N-2 hydroxyethylenepiperazine N-2 ethanesulfonic acid buffer (pH 8.0) containing 4 mM dithiothreitol, 2.5 mM pyridoxal phosphate and 25% polyvinylpolypyrrolidone. The preparation was homogenized and centrifuged at 12000 g for 15 min. One mL of the supernatant was placed in a 30 mL tube and 0.1 mL of 5 mM S-adenosyl methionine (AdoMet) was added. This was incubated for 1 h at 22°C. The 1-aminocyclopropane carboxylic acid formed was determined by its conversion to ethylene by the addition of 0.1 mL of 20 mM HgCl_2 _followed by 0.1 mL of 1:1 mixture of saturated NaOH/NaCl and incubated on ice for 10 min, and ethylene evolution was measured on a gas chromatograph. For control set AdoMet was not added. For ethylene evolution 5 mL of gas phase was removed with a syringe and ethylene was measured on a gas chromatograph (GC 5700, Nucon, New Delhi) equipped with 1.8 m *Porapack *N (80/100 mesh) column, a flame ionization detector and an integrator. Nitrogen was used as carrier gas. The flow rates of nitrogen, hydrogen and oxygen were 0.5, 0.5 and 5 mL s^-1^, respectively. The oven temperature was 100°C and detector was at 150°C. Ethylene identification was based on the retention time and quantified comparing with the peaks from standard ethylene concentrations.

### Data analysis

Data were analyzed statistically and standard error of the mean value was calculated. Analysis of variance was performed to identify the significant differences among treatments at P < 0.05 [28].

## Abbrevations

Adomet – S-adenosyl methionine; ACS – 1-aminocyclopropane carboxylic acid synthase; CA – carbonic anhydrase; C_i _– intercellular CO_2 _concentration; g_S _– stomatal conductance; P_N _– net photosynthetic rate

## Figures and Tables

**Table 1:** Effects of different concentrations of ethephon (2-chloroethyl phosphonic acid) applied at 30 d after sowing on the activity of 1-aminocyclopropane carboxylic acid synthase (ACS; (ng ACC kg^-1 ^leaf (FM) s^-1^) and ethylene evolution (ng kg^-1 ^leaf (FM) s^-1^) in two cultivars of mustard (*Brassica juncea*) at 15 d after the treatment. Values ± SE. Data followed by the same letter within a column are significantly not different.

Ethephon Treatments (mM)	High photosynthetic capacity cultivar (Varuna)		Low photosynthetic capacity cultivar (RH 30)	
		
	ACS	Ethylene	ACS	Ethylene
0	62.2 ± 5.4c	3.8 ± 0.2c	40.8 ± 3.6c	2.4 ± 0.2c
0.75	64.6 ± 5.8c	4.4 ± 0.3bc	44.4 ± 4.0c	3.2 ± 0.2bc
1.5	68.2 ± 5.8b	5.8 ± 0.3b	52.8 ± 4.6b	4.2 ± 0.3b
3.0	73.5 ± 6.0a	7.0 ± 0.5a	62.3 ± 5.6a	6.0 ± 0.4a
